# The root meristem growth factor *BrRGF6* positively regulates Chinese cabbage to infection of clubroot disease caused by *Plasmodiophora Brassicae*

**DOI:** 10.1093/hr/uhac292

**Published:** 2022-12-30

**Authors:** Wenjie Ge, Jing Zhang, Hui Feng, Yilian Wang, Ruiqin Ji

**Affiliations:** Liaoning Key Laboratory of Genetics and Breeding for Cruciferous Vegetable Crops, College of Horticulture, Shenyang Agricultural University, Shenyang, Liaoning 110866, China; Liaoning Key Laboratory of Genetics and Breeding for Cruciferous Vegetable Crops, College of Horticulture, Shenyang Agricultural University, Shenyang, Liaoning 110866, China; Liaoning Key Laboratory of Genetics and Breeding for Cruciferous Vegetable Crops, College of Horticulture, Shenyang Agricultural University, Shenyang, Liaoning 110866, China; Vegetable Research Institute, Liaoning Academy of Agricultural Sciences, Shenyang, Liaoning 110161, China; Liaoning Key Laboratory of Genetics and Breeding for Cruciferous Vegetable Crops, College of Horticulture, Shenyang Agricultural University, Shenyang, Liaoning 110866, China

## Abstract

Chinese cabbage has a high annual demand in China. However, clubroot disease caused by the infection of *Plasmodiophora brassicae* seriously affects its yield. Transcriptome analysis identified a root meristem growth factor 6 (*BrRGF6*) as significantly up-regulated in Chinese cabbage roots infected with *Plasmodiophora brassicae*. Quantitative reverse-transcription polymerase chain reaction and *in situ* hybridization analysis showed higher *BrRGF6* expression in susceptible materials than in resistant materials. After *Plasmodiophora brassicae* infection, *BrRGF6* expression was significantly up-regulated, especially in susceptible materials. Gene function analysis showed that the roots of *Arabidopsis* mutant *rgf6* grew faster than the wild-type, and delayed the infection progress of *Plasmodiophora brassicae*. A Protein, nuclear transcription factor Y subunit C (BrNF-YC), was screened from yeast two-hybrid library of Chinese cabbage induced by *Plasmodiophora brassicae*, and verified to interact with BrRGF6 by yeast two-hybrid co-transfer. Yeast one-hybrid and β-Glucuronidase activity analysis showed that BrNF-YC could directly bind to and strongly activate the promoter of *BrRGF6*. Transgenic verification showed that *BrRGF6* or *BrNF-YC* silenced Chinese cabbage significantly decreased the expression of *BrRGF6*, accelerated root development, and reduced incidence of clubroot disease. However, after overexpression of *BrRGF6* or *BrNF-YC*, the phenotype showed a reverse trend. Therefore, *BrRGF6* silencing accelerated root growth and enhanced resistance to clubroot disease, which was regulated by BrNF-YC.

## Introduction

Clubroot disease is a serious soil-borne disease of *Brassica rapa* L. ssp. *pekinensis*. Its typical symptom is the formation of galls in the roots of plants infected by *Plasmodiophora brassicae* [1]. As a result, plant growth is inhibited and some plants die, which seriously affects the quality and yield of Chinese cabbage [2]. Therefore, understanding the molecular regulation mechanism of host development after *Plasmodiophora brassicae* infection is important for the prevention and control of clubroot disease [3].

With the development of high-throughput sequencing technology, RNA sequencing (RNA-seq) is often used to analyze global transcriptome regulation and identify differentially expressed genes in different tissues, treatments, or stress conditions [4]. Analysis of gene expression changes in response to *Plasmodiophora brassicae* infection can provide a theoretical basis for the resistance mechanism to clubroot disease. In previous studies, RNA-seq showed that reactive oxygen species (ROS)-related genes were activated in resistant *Brassica oleracea* after inoculation with *Plasmodiophora brassicae* [5]. Genes related to auxin, disease resistance proteins, oxidative stress, and WRKY and MYB transcription factors of Chinese cabbage were identified as being involved in clubroot-disease resistance regulation [6]. Genes related to pathogen related molecular patterns, calcium influx, hormone signals, disease course related pathways, transcription factors, effector receptors and cell wall modification were identified as playing an important role in *Brassica rapa* during the early infection stage [7]. Although a large number of genes that play important roles in *Plasmodiophora brassicae* infection have been found by transcriptome analysis, the functions and regulatory mechanisms of most of them are unknown.

Plants suffer from stress caused by environmental factors, and to adapt to various environments, plants have evolved sensing and response systems, which uses highly sensitive signals to perceive the environment [8]. These signals are converted into cellular signals and then transduced to different cells and tissues to induce various reactions. Plants produce a variety of signaling molecules to mediate the response of specific cells to environmental stimuli [9]. Peptides are key factors in regulating plant growth and play important roles in plant growth, development, and stress responses. In recent years, scholars have identified a new gene family encoding small secretory peptides, also known as root meristem growth factors (*RGFs*), some called golven (*GLV*), and some are called clavata/embryo surrounding region like (*CLEL*). This family is encoded by 11 *RGF/CLEL/GLV* small signal peptide family genes with the same structure [10, 11]. Some of these proteins have been shown to control the maintenance of the root meristem, auxin flux, and gravity response [12]. RGF, as a secreted peptide, was considered a mediator of environmental stress in addition to plant hormones, and is a regulator of various aspects of plant growth [13].

There have been no reports on the relationship between RGFs and *Plasmodiophora brassicae* infection. In this study, transcriptomic analysis showed that the expression of a gene, *BrRGF6*, was significantly up-regulated in Chinese cabbage roots infected with *Plasmodiophora brassicae*. Investigation of the molecular mechanism suggested that *BrRGF6* plays a role in Chinese cabbage response to *Plasmodiophora brassicae* infection.

## Results

### Obtaining and Analyzing of *BrRGF6*

**Figure 1:**
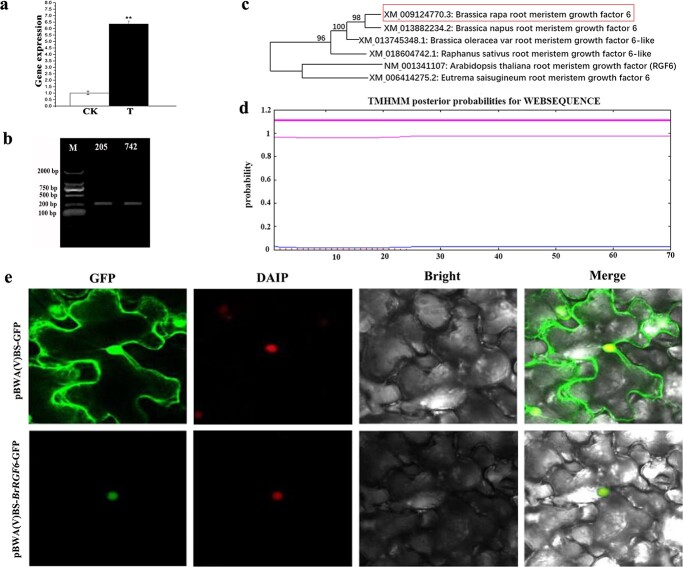
Obtaining *BrRGF6* and its subcellular location analysis. a Transcriptome data analysis of *BrRGF6*. CK, Expression level of *BrRGF6* in the control group root without inoculation. T, Expression level of *BrRGF6* in the group inoculated with *Plasmodiophora brassicae*. b Sequence Gel electrophoresis detection map of *BrRGF6.* M, D2000 Marker; 205, Fragments amplified in “SN205”; 742, fragments amplified in “SN742”. c Evolutionary tree of the candidate gene. d Analysis of transmembrane domain of *BrRGF6*. e Subcellular localization of *BrRGF6*. GFP: GFP fluorescence. DAPI: DAPI fluorescence. Bright: brightfield image. Merge: merged pictures of brightfield image, GFP fluorescence and and DAPI fluorescence. Three views were selected for electron microscopy observation.

A significantly up-regulated gene, *Bra040737*, was found in the diseased root of Chinese cabbage using RNA-Seq analysis (Fig. 1a). BLAST analysis of *Bra040737* showed that the entry number of the gene in NCBI was *XM_009124770.3*, and its open reading frame (ORF) length was 246 bp (encoding a peptide of 81 AA), which was located on chromosome A08. This gene had the highest homology with *RGF6* of multiple *Brassica* crops (Fig. 1c) Fig. 1b, and encodes a secreted peptide. Therefore, it was named *BrRGF6*. The full-length cDNAs cloned from “SN205” and “SN742” were both 246 bp (Fig. 1b) Fig. 1c, and their sequences were consistent with the reference sequence Fig. 1d.

TMHMM software analysis found that *BrRGF6* had no transmembrane domain (Fig. 1d). The results of subcellular localization assay showed that pBWA(V)BS-GFP carrier produced fluorescence signals in the nucleus, cell membrane, and cytoplasm of tobacco leaf cells. However, the pBWA(V)BS-*BrRGF6*-GFP carrier only produced fluorescence signal in the nucleus, and the fluorescence signal overlapped with DAPI nuclear staining (Fig. 1e). These results verified that *BrRGF6* was expressed in the nucleus, and a Y2H nuclear library could be used to screen its interacting proteins.

### Gene expression pattern analysis of *BrRGF6* in Chinese cabbage

**Figure 2:**
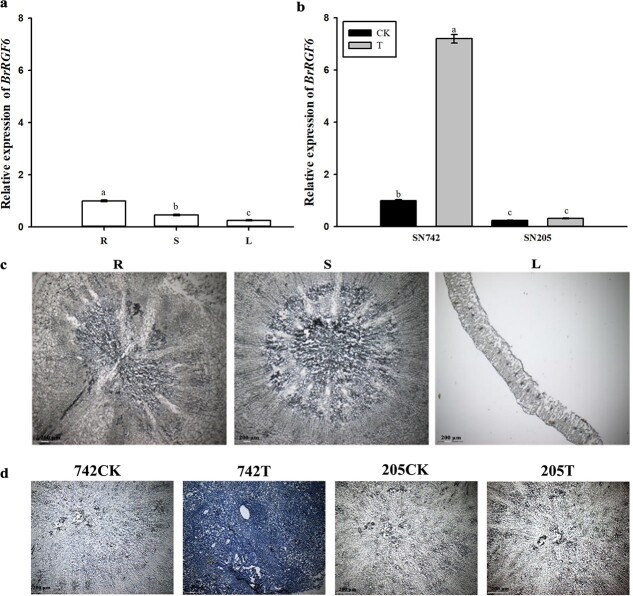
Expression pattern of *BrRGF6* in Chinese cabbage. a The expression of *BrRGF6* in *Plasmodiophora brassicae* uninoculated roots, stems, and leaves of “SN742”. p < 0.05, according to Duncan’s multiple range test. b The expression of *BrRGF6* in roots of “SN742” and “SN205” infected by *Plasmodiophora brassicae*. p < 0.05, according to Duncan’s multiple range test. Data shown represent mean ± SD (n = 3). c Hybridization signal of *BrRGF6* in uninfected roots, stems, and leaves of “SN742” R, root; S, stem; L, leave. d Hybridization signal of *BrRGF6* in roots of “SN742” and “SN205” infected by *Plasmodiophora brassicae*. CK, Control; T, Treatment with *Plasmodiophora brassicae* inoculation.

qRT-PCR analysis of *BrRGF6* in roots, stems, and leaves of “SN742” showed that its expression level was low in all tissues, although its expression was relatively higher in the roots (Fig. 2a). Expression pattern analysis of *BrRGF6* in the roots of “SN742” and “SN205” before and after infection by *Plasmodiophora brassicae* showed that *BrRGF6* expression was up-regulated after inoculation with *Plasmodiophora brassicae* (Fig. 2b). Interestingly, the expression of *BrRGF6* in the *Plasmodiophora brassicae-*treated susceptible material “SN742” was significantly enhanced, and its expression level in resistant materials was lower than that in susceptible ones, which suggested that *BrRGF6* plays an important role in Chinese cabbage response to infection by *Plasmodiophora brassicae*.

*In situ* hybridization analysis was used to further verify the specific expression of *BrRGF6*. The results showed that there was no obvious hybridization signal in all tissues of uninoculated “SN742” (Fig. 2c), which verified the results of qRT-PCR and indicated that the expression of *BrRGF6* was relatively low in uninfected Chinese cabbage. *In* situ hybridization analysis of the expression pattern of *BrRGF6* in the roots of *Plasmodiophora brassicae*-infected “SN742” and “SN205” revealed an obvious blue hybridization signal in *Plasmodiophora brassicae-*treated “SN742” (Fig. 2d), which was consistent with the results of qRT-PCR, and further confirmed the conclusion that *BrRGF6* expression was up-regulated in susceptible material in response to *Plasmodiophora brassicae* infection.

### Resistance identification in *Arabidopsis* mutant *rgf6*

**Figure 3:**
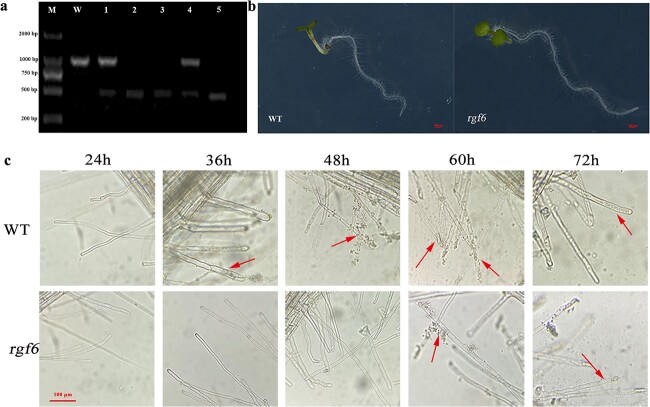
Resistance identification of *Arabidopsis* mutant *rgf6* after inoculation with *Plasmodiophora brassicae*. a The identification results of *Arabidopsis* mutant *rgf6* by “Tri-Primer-PCR”. M, D2000 bp marker; W, Wild-type of *Arabidopsis*; 1–5, different plants of *Arabidopsis* mutant *rgf6*. b Observation of WT and *rgf6* phenotypes. c Infection of *Arabidopsis* WT and mutant *rgf6* after inoculation with *Plasmodiophora brassicae.* The arrow points to the infection site of *Plasmodiophora brassicae* spores.

To identify the function of *BrRGF6*, we screened the *Arabidopsis* homozygous mutant *rgf6*, and investigated its resistance to clubroot disease. The results showed that only a 1100 bp LP + RP band could be identified the WT plants; mutant 1 and 4 showed two PCR products at 1100 bp (LP + RP) and 550–600 bp (BP + RP) respectively, indicating that they were heterozygous mutants, while mutants 2, 3, and 5 only showed a 550–600 bp BP + RP fragment, identifying them as homozygous mutants (Fig. 3a).

qRT-PCR showed that the expression of *AtRGF6* in homozygous offspring of *rgf6* was significantly lower than that in the WT (Fig. S1) Fig. 1S, this indicated that T-DNA insertion had resulted in the mutation and function loss of *RGF6* in *Arabidopsis*. Comparing the phenotypes of the roots of mutant *rgf6* and WT, we found that the roots of the *rgf6* mutant grew faster and contained thicker root hairs than that of the WT (Fig. 3b). At 36 h after inoculation with *Plasmodiophora brassicae*, an enormous number of *Plasmodiophora brassicae* zoospores were attached to the root hair epidermis of WT, and *Plasmodiophora brassicae* spores began to invade the root hairs at 48 h after inoculation. However, it required 60 h for the zoospores to attach to the root hairs epidermis of *rgf6* after inoculation, and a large number of spores invaded root hairs of *rgf6* at about 72 h after inoculation (Fig. 3c). Compared with WT plants, the pathogenic process of *Plasmodiophora brassicae* was delayed in *rgf6.* The above results showed that the absence of *RGF6* could inhibit *Plasmodiophora brassicae* infection by promoting the growth and development of the roots.

**Figure 4:**
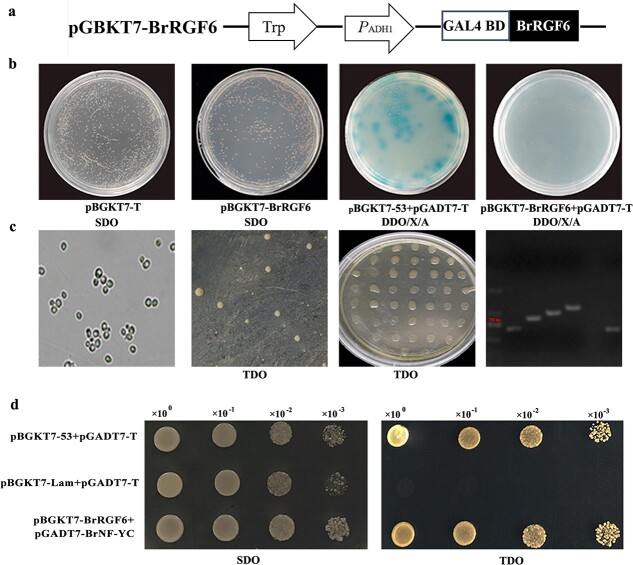
Interacting proteins screening of BrRGF6 from yeast two-hybrid nuclear library. a Structure diagram of pBGKT7-BrRGF6 in the yeast two-hybrid analysis. b Toxicity testing and self-activation detection of pBGKT7-BrRGF6. SDO, SD/−Trp; DDO/X/A, SD/−Trp/−Leu/X-a-gal/AbA. c Screening of yeast two hybrid library. From left to right: Observation on the combination of bait and Y187 library bacterial liquid; Colony growth on SD/−Trp/−Leu (DDO); Colony growth on SD/−Trp/−Leu/-His (TDO); PCR identification of positive clones (part). d Yeast two hybrid co-transformation verification. pGADT7-T7 + pGBKT7–53 as the positive control; pGADT7-T7 + pGBKT7-Lam as the negative control.

### Screening interacting proteins of BrRGF6 from yeast two-hybrid nuclear library

To obtain proteins that might interact with BrRGF6, the bait vector pGBKT7-BrRGF6 was constructed (Fig. 4a). After testing, it was found that yeast strains containing pGBKT7-BrRGF6 and pGBKT7-T were white, and showed the same growth trend on SDO plates, thus pGBKT7-BrRGF6 has no toxicity. On DDO/X/A plates, the colonies containing pGBKT7–53 + pGADT7-T were blue, but the colonies containing pGBKT7-BrRGF6 + pGADT7-T were white, thus indicating that pGBKT7-BrRGF6 cannot produce auto-activation (Fig. 4b). Thus, pGBKT7-BrRGF6 can be used for Y2H library screening. Typical cloverleaf-like binders were visible under the microscope when single Y2H colony carrying pGBKT7-BrRGF6 was fused with the Y187 yeast nuclear library (Fig. 4c). After primary screening, 148 colonies grew on TDO (SD/−Trp/−Leu/-His). After secondary screening, a total of 34 colonies grew on TDO plates (SD/−Trp/−Leu/-His). Electrophoresis detection showed that the length of the inserted fragments ranged from 500–1500 bp (Fig. 4c). Among them, 24 positive clones were successfully sequenced and annotated to five proteins (Table 1). Among them, 19 clones were annotated as *B. rapa* nuclear transcription factor Y subunit C (BrNF-YC); two clones were annotated to *B. rapa* N-(5′-phosphoribosyl) anthranilate isomerase 1, and other clones were only annotated to one protein each, respectively. Therefore, the transcription factor BrNF-YC should be a key interaction protein of BrRGF6.

To verify the interaction between BrNF-YC and BrRGF6, the recombinant vector pGADT7-BrNF-YC for Y2H co-transfer was constructed. The results of co-transfer of pBGKT7-BrRGF6 and pGADT7-BrNF-YC showed that the clones of positive control (pGADT7-T7 + pGBKT7–53), negative control (pGADT7-T7 + pGBKT7-Lam), and experimental group (pGADT7-BrNF-YC + pBGKT7-BrRGF6) at each dilution concentration could grow normally on the SD/−Trp/−Leu (DDO) plate, indicating successful yeast transformation. On the SD/−Trp/−Leu/-His (TDO) plate, the negative control could not grow. However, the co-transferred clones of the experimental group and the positive control grew normally (Fig. 4d).

### The binding and regulatory relationship between BrNF-YC and the promoter of *BrRGF6* (*PBrRGF6*)

**Figure 5:**
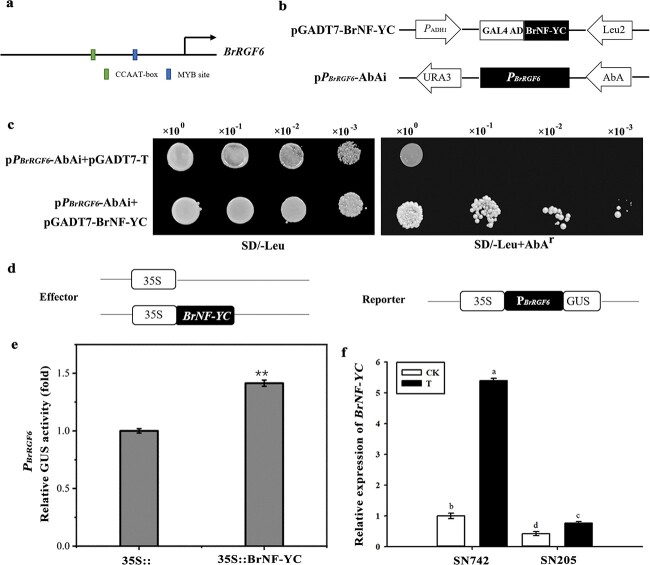
Binding and regulatory relationship between BrNF-YC and *PBrRGF6*. a Cis elements analysis in the promoter of *BrRGF6*. b Schematic diagram of the vector structure used in the yeast one-hybridization (Y1H) analysis. c Y1H analysis. pGADT7-BrNF-YC as the prey, p*PBrRGF6-*AbAi as the bait, and pGADT7-T as a negative control. d Structure diagram of effector and reporter constructs used in the GUS activity analysis. e GUS activity analysis of *PBrRGF6*. Three independent transfections were analyzed. p < 0.05, according to a t-test. f The expression of *BrNF-YC* in roots of “SN742” and “SN205” infected by *Plasmodiophora brassicae*. CK, Control; T, Treatment with *Plasmodiophora brassicae* inoculation. p < 0.05, according to Duncan’s multiple range test. Data shown represent mean ± SD (n = 3).

To further study the regulatory relationship between BrNF-YC and *BrRGF6*, the 1041 bp promoter of *BrRGF6***(***PBrRGF6*) was amplified, and certain transcription elements such as MYB and TATA-Boxes were found in *PBrRGF6* using the Plantcare online software. Especially, an NF-YC specific binding element (CCAAT-Box) was found at 490 bp upstream of the initiation codon of *BrRGF6* (Fig. 5a).

To investigate whether BrNF-YC can specifically bind to *PBrRGF6*, vector p*P_BrRGF6_-*AbAi for Y1H was constructed (Fig. 5b). The minimum inhibitory concentration of AbA for Y1H-p*P_BrRGF6_-*AbAi was screened using SD/-Ura/AbA (50–500 ng/mL), and found that SD/-Ura/AbA (150 ng/mL) was appropriate. The results of Y1H showed that the yeast colonies containing p*P_BrRGF6_-*AbAi + pGADT7-BrNF-YC and p*P_BrRGF6-_*AbAi + pGADT7-T could grow normally on SD/−Leu medium, which indicated that pGADT7-T and pGADT7-BrNF-YC were successful transformed into Y1H-p*P_BrRGF6_-*AbAi yeast. However, on SD/−Leu/AbA (150 ng/mL) at different dilution concentrations, only Y1H-p*P_BrRGF6_-*AbAi + pGADT7-BrNF-YC could grow normally. These results verified that BrNF-YC can directly and specifically bind to *PBrRGF6* (Fig. 5c).

The regulatory mode of BrNF-YC on *PBrRGF6* was detected using GUS activity analysis, pRI-BrNF-YC and pBI-*PBrRGF6* were constructed (Fig. 5d). The result showed that the GUS activity of *PBrRGF6* was enhance by co-transformation with the strains containing pRI101-BrNF-YC, compared with those containing the empty vector (Fig. 5e), indicating that BrNF-YC activated the *PBrRGF6*.

The qRT-PCR results showed that *BrNF-YC* was strongly induced by *Plasmodiophora brassicae* infection in “SN742” (Fig. 5f). The relative expression trend of *BrNF-YC* was roughly consistent with that of *BrRGF6* after infection by *Plasmodiophora brassicae* (Fig. 2b). Therefore, *BrNF-YC* can respond *Plasmodiophora brassicae* infection by specifically binding the promoter region of *BrRGF6* to positively regulate its expression.

### Effects of *BrRGF6* and *BrNF-YC* silence on the infection progress of *Plasmodiophora brassicae* in Chinese cabbage

**Figure 6:**
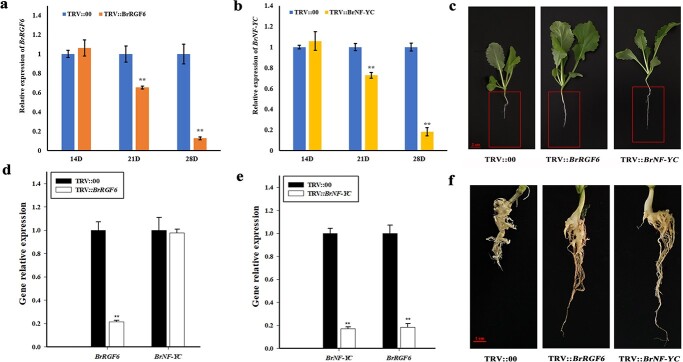
Silencing efficiency of *BrRGF6*/*BrNF-YC* and the resistance of *BrRGF6*/*BrNF-YC* silencing Chinese cabbage to clubroot disease. a Silencing efficiency of *BrRGF6.* p < 0.05, according to t-test. b Silencing efficiency of *BrNF-YC.* p < 0.05, according to t-test. c Root growth observation of the silenced lines. d Analysis of genes expression in TRV::*BrRGF6* silenced plants. p < 0.05, according to t-test. e Analysis of genes expression in TRV::*BrNF-YC* silenced plants. p < 0.05, according to t-test. Data shown represent mean ± SD (n = 3). f Clubroot disease symptom of VIGS silenced plants infected by *Plasmodiophora brassicae*.

To further study the role of *BrRGF6* and *BrNF-YC* in response to *Plasmodiophora brassicae* infection and their interaction relationship, pTRV2-*BrRGF6* and pTRV2-*BrNF-YC* recombinant vectors were constructed (Fig.S2) Fig. 2S. The gene silencing efficiency was analyzed at 14^th^,21^st^, and 28^th^ days after VIGS treatment. Compared with TRV::00, the expression of *BrRGF6* and *BrNF-YC* began to decrease on the 21^st^ day after treatment, and the decline was more obvious on the 28^th^ day (Fig. 6a, b). Therefore, the plants were inoculated with *Plasmodiophora brassicae* on the 21^st^ day after VIGS treatment. Further, the expression of *BrRGF6* was decreased significantly in TRV::*BrNF-YC* silenced lines, while the expression of *BrNF-YC* was not decreased significantly in the TRV::*BrRGF6* silenced lines (Fig. 6d, e). Consistent with this, the expression of *AtNF-YC* was not significant changed in *Arabidopsis rgf6* (Fig. S1) Fig. 1S. These results indicated that *BrNF-YC* regulates the expression of *BrRGF6*.

When the clubroot symptoms appeared, the clubroot disease index was investigated. The results of clubroot disease index showed that the incidence of infection in the TRV::00 groups was 70.00%. While the incidences in the TRV::*BrRGF6* and TRV::*BrNF-YC* groups were significantly reduced, to 36.67% and 36.11% respectively. The disease indexes of the TRV::00, TRV::*BrRGF6*, and TRV::*BrNF-YC* groups were 43.89, 17.78, and 19.99, respectively (Table S2). The incidence in the TRV::*BrRGF6* and TRV::*BrNF-YC* groups were significantly lower than that of the TRV::00 groups (Fig. 6f). These results indicated that *BrRGF6* or *BrNF-YC* silenced Chinese cabbage displayed enhanced the resistance to clubroot disease.

Observation of the phenotypes of the silenced plants showed that the TRV::*BrRGF6* or TRV::*BrNF-YC* groups was growth better than the empty vector TRV::00 group. The root length of TRV::*BrRGF6* or TRV::*BrNF-YC* group was significantly higher than that of TRV:: 00 group (Fig. 6c, Table. S3), which indicated that silencing of *BrRGF6* or *BrNF-YC* in Chinese cabbage could promote root growth. Therefore, we speculated that *BrNF-YC* can regulate the expression of *BrRGF6*, and both of them play an important role in the response to infection of *Plasmodiophora brassicae* by promoting root growth.

### Effects of *BrRGF6* and *BrNF-YC* transient overexpression on the infection progress of *Plasmodiophora brassicae* in Chinese cabbage

**Figure 7:**
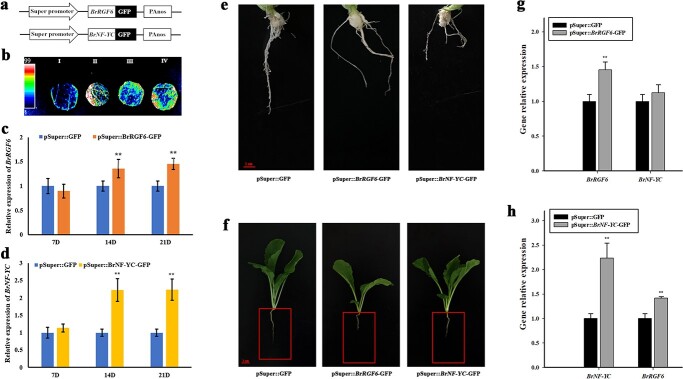
The Effection of *BrRGF6* and *BrNF-YC* overexpression on the progress Chinese cabbage infected by *Plasmodiophora brassicae*. a Structure diagram of pSuper::*BrRGF6*-GFP and pSuper::*BrNF-YC*-GFP recombinant vectors in transient overexpression. b Observation of fluorescence signals in leaves of transient overexpression plants. I, Leaves of untreated Chinese cabbage; II, Leaves of pSuper::GFP transient overexpression plants; III, Leaves of pSuper::*BrRGF6*-GFP transient overexpression plants; IV, Leaves of pSuper::*BrNF-YC*-GFP transient overexpression plants. c Efficiency of *Agrobacterium tumefaciens* mediated pSuper::*BrRGF6*-GFP transient transformation*.* d Efficiency of *A. tumefaciens* mediated pSuper::*BrNF-YC*-GFP transient transformation*.* e Clubroot disease symptom of transient overexpression plants infected by *Plasmodiophora brassicae* f Root growth observation of the transient overexpression plants. g Analysis of genes expression in pSuper::*BrRGF6*-GFP transient overexpression plants. h Analysis of genes expression in pSuper::*BrNF-YC*-GFP transient overexpression plants. p < 0.05, according to t-test. Data shown represent mean ± SD (n = 3).

pSuper::*BrRGF6*-GFP and pSuper::*BrNF-YC*-GFP recombinant vectors was constructed (Fig. 7a). The leaves of overexpression treated plants showed obvious GFP signals, which proved that the transformation was successful (Fig. 7b). qRT-PCR found that the expression *BrRGF6* and *BrNF-YC* was significantly increased on the 14^th^ d after overexpression transient transformation (Fig. 7c-d). Therefore, overexpressed plants were inoculated with *Plasmodiophora brassicae* on the 14^th^ day after the transient transformation treatment. After 20 days of inoculation, it was found that most of the plants had root swelling symptoms. The results of clubroot disease index showed that the incidence of infection in the pSuper::GFP group was 64.00%. While the incidences in the pSuper::Br*RGF6*-GFP and pSuper::Br*NF-YC*-GFP groups increased significantly, to 71.91% and 70.00%, respectively. The disease indexes of pSuper::GFP, pSuper::*BrRGF6*-GFP and pSuper::*BrNF-YC*-GFP groups were 24.85, 42.59 and 37.77 respectively (Table S4). The disease symptom of pSuper::*BrRGF6*-GFP and pSuper::*BrNF-YC*-GFP was more obvious than that of pSuper::GFP (Fig. 7e).

The phenotype observation of overexpression plants found that the root length of *BrRGF6* overexpressed plants (pSuper::*BrRGF6*-GFP treatment groups) was significantly shorter than that of control plants (pSuper:GFP treatment groups) (Table S5, Fig. 7f). However, the roots length of *BrNF-YC* overexpressed plants (pSuper::*BrNF-YC*-GFP treatment groups) have not significant difference with control plants (pSuper:GFP treatment groups). Therefore, we speculate that *BrRGF6* is a key gene regulating root growth. Otherwise, the expression of *BrRGF6* was significantly increased in *BrNF-YC* overexpression plants, while the expression of *BrNF-YC* was not significantly increased in *BrRGF6* overexpression plants (Fig. 7g-h). Therefore, it suggested that *BrNF-YC* played its role by regulating the expression of *BrRGF6*.

## Discussion

A series of changes occur when plants are infected by pathogens, such as innate immune response activation of pathogen-associated molecular pattern (PAMP) triggering immunity [25]. Plant recognition of pathogens leads to the production of reactive oxygen that activates defense responses [26], and changes in protein/gene expression related to the course of disease [27, 28]. Transcriptomic analysis revealed that genes related to PAMPs, calcium influx, hormone signaling, disease course correlation pathways, transcription factors, effector receptors, and cell wall modification were significantly up-regulated after infection by *Plasmodiophora brassicae* [7, 29, 30]. In this study, we found that the expression level of *BrRGF6* in “SN205” roots was little changed after infection by *Plasmodiophora brassicae*, while that in “SN742” roots was increased significantly after infection (Fig. 2, 3). This showed that *BrRGF6* expression is sensitive to *Plasmodiophora brassicae* in susceptible materials. The expression of this gene in resistant materials is low and was not affected by *Plasmodiophora brassicae*. We speculated that the up-regulated expression of this gene was caused by the accumulation of pathogenic bacteria in the roots. Investigation of resistance in *Arabidopsis rgf6* showed that knockout of the gene delayed the infection time of *Plasmodiophora brassicae* (Fig. 4c). VIGS experiments showed that the disease index of TRV::*BrRGF6* silenced strain was lower than that of TRV::00 (Fig. 6). This suggested that the gene might promote the occurrence of the disease.

**Table 1:** Blast analysis results of BrRGF6 candidate interacting protein

**Type code**	**Description of interacting protein**	**Repeated times of screening**	**Accession**	**Identity**
INT-1	*Brassica rapa* nuclear transcription factor Y subunit C (*BrNF-YC*)	19	XM_009151558.3	99%
INT-2	*B. rapa* N-(5′-phosphoribosyl) anthranilate isomerase 1	2	XM_009127362.3	97%
INT-3	*B. rapa* 60S ribosomal protein L22–2	1	XM_009136583.2	99%
INT-4	*Brassica napus* isocitrate dehydrogenase [NAD] catalytic subunit 5	1	XM_013876977.2	99%
INT-5	*B. rapa* threonine synthase 2	1	XM_009129703.3	98%

Previous reports found that r*gf6/glv1/clel6* and its homolog *glv2* are expressed in the outer cell layer of the hypocotyl, preferentially in the region of rapid cell elongation, and regulate the auxin gradient of *Arabidopsis* hypocotyls [31]. Its main function is to regulate plant gravitropism and lateral root development. When treated with synthetic peptides containing the C-terminal conserved sequence of the RGF6 protein precursor or overexpression of *RGF6*, *Arabidopsis* roots showed irregular wavy roots and deletions [11]. *RGF6* overexpression delayed lateral root development [32], and regulated lateral root development independently of auxin pathways, such as IAA. Treatment of poplar seedlings with CLEL6 peptides will cause roots to thicken and form abnormal lateral roots, which in many cases form clusters. This root thickening was mainly caused by the increase of epidermis, hypodermis, and cortical cells [29]. Compared with the WT root tip meristem, CLEL6 (RGF6) peptide induced a long root tip apical meristem (RAM) to expand and increase cell number, and *GLV1* (*RGF6*) overexpression delayed lateral root development [31]. In this study, the root growth of the *Arabidopsis rgf6* mutant was faster than that of the WT, and the lateral root hairs were thicker (Fig. 4b). Furthermore, the growth potential of TRV::*BrRGF6* silenced line was stronger and the root growth was faster than that of the TRV::00 group (Fig. 6c), and the root growth of *BrRGF6* overexpression plants was inhibited (Fig. 7f). Therefore, we speculated that *BrRGF6* accelerates *Plasmodiophora brassicae*’s invasion into epidermal and cortical cells by affecting root growth.

An important approach to determine intracellular signal transduction is to study protein interactions [33]. The Y2H system can directly and accurately reveal the interaction between two proteins in an organism by detecting the expression of reporter genes [34]. In this study, a highly reproducible interacting protein (BrNF-YC) of BrRGF6 was screened using a cDNA library of Chinese cabbage roots (Table. 1) (Fig. 4), indicating a close interaction with BrRGF6. Verification by Y2H co-transformation showed an interaction between BrRGF6 and BrNF-YC in Chinese cabbage infected by *Plasmodiophora brassicae* (Fig. 4d, e). However, when analyzing the expression pattern of *Arabidopsis* homologous mutant *rgf6*, it was found that the expression of *AtNF-YC* did not change significantly in response to decreased *AtRGF6* expression (Fig. S1) Fig. 1S. Therefore, we conducted further research on BrNF-YC. Previous studies have found that NF-Y is a nuclear protein that binds the CCAAT sequence in gene promoters with very high specificity. The CCAAT-box is a regulatory element that is generally located at a conserved distance of 60–100 bp from the transcription start site, and it exists in 25% of eukaryotic promoters [35]. NF-Y often exists as a gene family in plants, including NF-YA, NF-YB, and NF-YC [36]. NF-Y functions as a dimer or trimer. Initially, NF-YB and NF-YC are dimerized in the cytoplasm and then integrate into the nucleus, aggregating NF-YA components and subsequently binding to DNA and affecting transcription [37]. The functional analysis of *BrNF-YC* by VIGS showed that the expression of *BrRGF6* in the silenced strain also decreased (Fig. 6d). NF-YC is a nuclear transcription factor, and cis-element binding site (CCAAT-box) was found at position 490 bp in the upstream promoter region of *BrRGF6* (*PBrRGF6*) (Fig. 5a); therefore, we speculated that BrNF-YC binds to *PBrRGF6* to regulate *BrRGF6* expression*.* Yeast-one hybridization and GUS activity analysis confirmed that *BrNF-YC* could bind to *PBrRGF6* and positively regulated its activity (Fig. 5). The positive regulation was confirmed using qRT-PCR. These results further confirmed that upregulation of *BrNF-YC* expression caused a significant up-regulation of *BrRGF6* expression.

**Figure 8:**
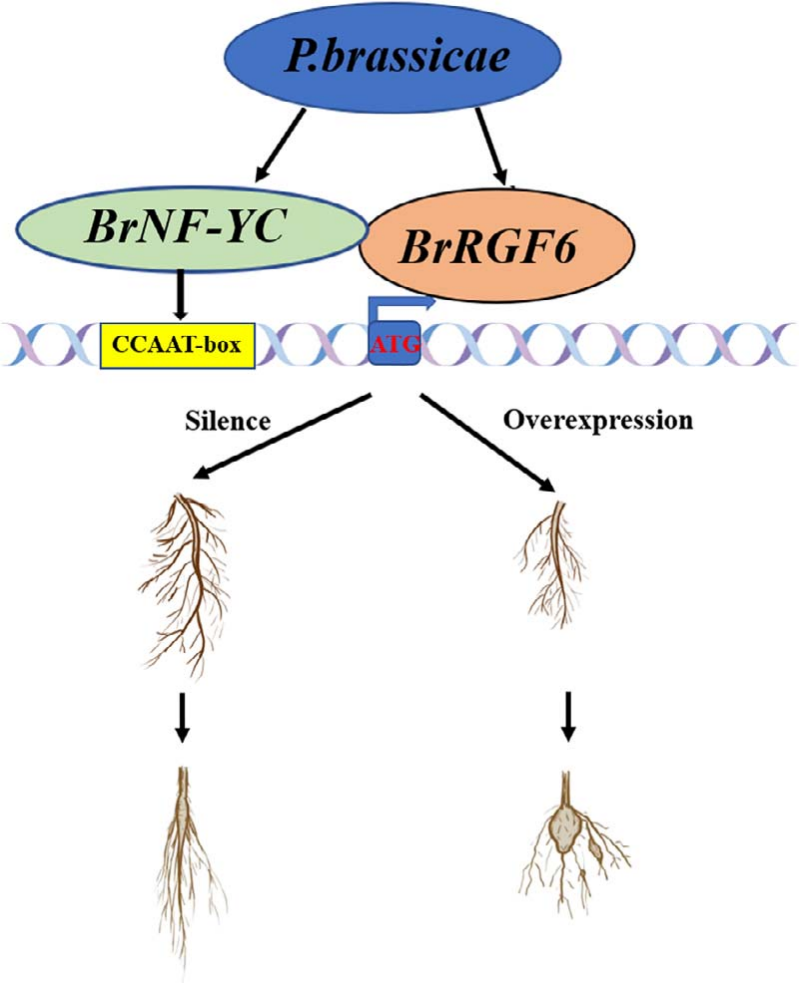
Proposed regulation model of *BrRGF6* and *BrNF-YC* in the response of Chinese cabbage responsed to infection by *Plasmodiophora brassicae*. BrNF-YC can interact with BrRGF6 to be infected by *Plasmodiophora brassicae* infection and directly bind to and strongly activate the promoter of *BrRGF6*. Silence of *BrRGF6* and *BrNF-YC* can accelerate root development and reduce the incidence rate of clubroot disease. However, the opposite trend was observed after overexpression of *BrRGF6*.

NF-YC subunits have been implicated in legume-*rhizobium* interactions [38]. Studies have shown that PvNF-YC1 might regulate *rhizobium* infection and nodule development by regulating cell cycle genes [39]. However, research on the defensive role of BrNF-YC in the process of Chinese cabbage infection of *Plasmodiophora brassicae* has not been reported, nor has the role of BrRGF6. This study revealed that the expression of *BrRGF6* and *BrNF-YC* changed significantly after *Plasmodiophora brassicae* inoculation (Fig. 2b and Fig. 5e), indicating that they respond to certain regulatory mechanisms during the process of *Plasmodiophora brassicae* infection*.*

Previously, researchers pointed out that NF-Y is involved in the regulation of root system and hypocotyl development [40], as well as the interaction between plants and microorganisms, and plant and environment [41, 42]. RGF6 microsecretory peptide also regulates the hypocotyl, lateral root development, and geotropism [10, 11]. Our study found that the root growth and development of *Arabidopsis* mutants and VIGS lines were faster than those of the control, and their disease resistance was enhanced, while the clubroot-disease resistance of transient overexpression Chinese cabbage was significantly reduced (Fig. 3, Fig. 6 and Fig. 7), suggesting that root growth is closely related to disease resistance. However, the interaction between BrNF-YC and BrRGF6 has not been reported so far. This study confirmed the interaction between them through Y2H, Y1H, GUS activity analysis, and VIGS assays. *BrNF-YC* and *BrRGF6* play an important role in the regulation of plant root morphogenesis, growth, and development. In addition, *BrRGF6* was positively regulated by the transcription factor *BrNF-YC*. Combined with previous research reports, we speculated that *BrRGF6* and *BrNF-YC* regulates the root structure of plant tissues, affects root tip cell differentiation and hypocotyl elongation, and has a certain influence on infection efficiency of *Plasmodiophora brassicae*, including altering the speed of infection. Finally, it was concluded that *BrRGF6* silencing led to accelerated root growth and enhanced resistance to clubroot disease, which may be regulated by BrNF-YC (Fig. 8).

## Materials and methods

### Plant materials

The plant materials used in this experiment were the resistant variety “SN205” and susceptible varieties “SN742” and “ECD05” of Chinese cabbage. *Plasmodiophora brassicae* was the *no. 4* physiological race, which has been isolated and identified from clubroots of Chinese cabbage in previous studies [14]. The seeds of Colombian wild-type *Arabidopsis* “WT” and tobacco (*Nicotiana Benthamiana*) were provided by the vegetable genetics and breeding laboratory of Shenyang Agricultural University, Shenyang, China. The *Arabidopsis* mutant *rgf6* (*SALK_133489*) was purchased from the *Arabidopsis* Biological Resource Center (ABRC; https://abrc.osu.edu/).

### Acquisition of test materials

Soil with *Plasmodiophora brassicae* was prepared according to previous methods [15]. The germinating seeds of “SN205” and “SN742” were seeded in 5-cm-diameter pots containing the soil with *Plasmodiophora brassicae* separately, and then grown in a greenhouse at 25°C, with 70 to 90% soil moisture. An uninoculated control group was set at the same time. Samples collected at the time of obvious root swelling in the inoculated plants were used as materials, uninoculated plants in the same period were used as the control. RNA was extracted from root tissues using an RNApure Ultrapure Total RNA Rapid Extraction Kit (Aidlab, Beijing, China). cDNAs were then obtained using a Tianscript II cDNA first strand synthesis Kit (Tiangen, Beijing, China).

### Obtaining and Analyzing the target gene sequence

A candidate gene was selected by RNA-seq analysis, and was found to be significantly up-regulated in roots infected by *Plasmodiophora brassicae*. A pair of specific primers (*BrRGF6*-F/R) for target gene were designed using a reference cDNA sequence that was obtained from the Chinese cabbage database (http://brassicadb.cn/#/) (the primers are shown in Table S1).

The roots cDNAs of “SN205” and “SN742” at the four true leaf stage were used as the template for PCR amplification of the full-length cDNA. Gel electrophoresis was performed using 2% agarose and the amplicon were purified using a Gel Extraction Kit (CWBIO, Jiangsu, China). The full-length *BrRGF6* cDNA sequences of “SN205” and “SN742” were obtained by sequencing (Sangon Biotech, Shanghai, China) and used for sequence alignment. BLAST analysis of gene homologous sequence was performed at the NCBI (https://www.ncbi.nlm.nih.gov/). Signal peptide prediction was performed using the SignalP server (http://www.cbs.dtu.dk/services/SignalP). TMHMM (http://www.cbs.dtu.dk/services/TMHMM/) was used to predict the membrane structure domain of *BrRGF6*.

### Subcellular localization analysis

The *BrRGF6* coding sequence with the termination codon removed was amplified (primers in Table S1) and cloned into the *Hind*III and *Bam*HI sites of vector pBWA(V)BS-GFP vector to obtain the recombinant vector pBWA(V)BS-*BrRGF6*-GFP. pBWA(V)BS-*BrRGF6*-GFP and pBWA(V)BS-GFP were transferred separately into *Agrobacterium tumefaciens* strain GV3101. After culture to OD600 = 0.5–0.6, the strains were centrifuged at 700 × *g* for 5 min to collect the cell pellet. The cells were resuspended in buffer (1 M MgCl_2_, 100 mM acetosyringone, and 1 M MES, pH 5.6) at an OD600 of 1.0 and injected into 4-week-old tobacco leaves. The tobaccos were cultured in the dark for 24 h and in light for 24–48 h at room temperature. Before observation, the leaves were stained using 4′,6-diamidino-2-phenylindole (DAPI) (Coolaber, Beijing, China) for 10 minutes, and then washed twice with phosphate buffered saline (PBS) (NaCl 137 mmol/L, KCl 2.7 mmol/L, Na_2_HPO_4_ 4.3 mmol/L, KH_2_PO_4_ 1.4 mmol/L, PH 8), for 5 minutes each time. GFP fluorescence was observed under a Confocal laser microscope (TCS SP8, Leica, Wetzlar, Germany).

### Quantitative reverse-transcription polymerase chain reaction (qRT-PCR)

Specific primers for qRT-PCR of *BrGRF6* (*BrRGF6*-qPCR-F/R) was designed, and the *BrActin* gene was used as the internal control [16] (the primers are shown in Table S1). The expression patterns of *BrGRF6* were studied in the roots, stems, and leaves of 14-day-old uninoculated “SN742”, and the expression patterns of *BrGRF6* a were studied using the root cDNA of “SN742” and “SN205” after inoculation by *Plasmodiophora brassicae*. The fluorescence quantification kit UltraSYBR Mixture (Low ROX) was used for the qPCR step, and the analyses were performed using the QuantStudio6 Real Time PCR System (ThermoFisher, Waltham,MA, USA). Analysis of the relative gene expression data was performed using the 2^−ΔΔCt^ method [17], and SigmaPlot 12.5 (Systat Software, Inc., San Jose, CA, USA) was used to make graphs. Three replicates were performed for each different treatment.

### *In situ* hybridization

Three replicates of the roots, stems and leaves of “SN742” and the roots of “SN742” and “SN205” after inoculation by *Plasmodiophora brassicae* were used for *in situ* hybridization experiments. Samples were fixed in 4% paraformaldehyde (PFA; RNase-free) solution, dehydrated in an EtOH series, cleared using dimethylbenzene, and embedded in paraffin. The detailed test steps were carried out according to the description by Pawlowski [18]. The embedded samples were cut into 10 μm slices using a frozen slicer (Leica, Cm1850uv) for subsequent hybridization [19].

The cDNA sequence of *BrRGF6* was amplified using primers *BrRGF6*-situ-F/R (Table S1) and cloned into the *Hind*III and *Bam*HI sites of vector pSPST18 to construct recombinant vector *BrRGF6-*pSPST18 using a ClonExpress II One Step Cloning Kit (Vazyme, Nanjing, China). *BrRGF6-*pSPST18 was then digested using *Hind*III and *Bam*HI (NEB, Beijing, China), respectively. *In vitro* transcription was then performed using digoxigenin (DIG) labeling with a SP6/T7 Transcription kit (Roche, Basel, Switzerland). The sections (10 μm thick) were hybridized with the specific DIG-labeled RNA probes according to the instructions of the kit (DIG RNA Labeling Kit (SP6/T7), Roche), and then observed and photographed under a microscope (Nikon Eclipse 80i, Tokyo, Japan).

### Resistance identification of *Arabidopsis* mutants *rgf6*

The seeds of *Arabidopsis* mutant “*rgf6*” and “WT” (Columbia-0) were washed with sterile water and then with 70% ethanol for 1 min. They were then sterilized using 0.1% mercury for 10 minutes and washed six times with sterile ddH_2_O in Clean Bench cabinets. The seeds were seeded in 1/2 Murashige and Skoog (MS) medium and placed in refrigerator for 3–4 days for vernalization at 4°C. The plants were then grown in 1/2 MS medium for 15 days before being transferred to the medium with peat, vermiculite, and perlite (3:2:1, vol/vol/vol) and kept in a growth chamber (22°C, 16 h light / 8 h dark). The homozygous “*rgf6*” plants were selected using leaf DNAs from “*rgf6*” and “WT” by Tri-Primer-PCR with primers of LP, RP, and LB (Table S1) obtained from the SIGnAL website (http://signal.salk.edu/tdnaprimers.2.htmL). Homozygous mutants were cultured until the offspring seeds were obtained. The expression of *AtRGF6* was detected in 14-day-old seedling-sized plants of “*rgf6*” and “WT” using a pair of specific primers (*AtRGF6*-qPCR in Table S1) for *AtRGF6* by qRT-PCR. The 18S *rRNA* gene was used as the internal control. The offspring seeds of homozygous “*rgf6*” and “WT” were seeded on 1/2 MS medium, and the phenotype of their roots was observed in the seedlings with two true leaves under a stereomicroscope (Model SMZ25, Nikon, Tokyo, Japan).

The sterilized offspring seeds of homozygous “*rgf6*” and “WT” were seeded in petri dishes containing moist filter paper. When the plants grew two true leaves, a 10 [7]/mL *Plasmodiophora brassicae* suspension was sprayed on the roots for inoculation [20]. Five randomly selected plants from the inoculated “*rgf6*” and “WT” were observed under a compound microscope (Eclipse 80i; Nikon, Tokyo, Japan) at 12 h intervals.

### Screening for interacting proteins of BrRGF6 using the yeast two-hybrid (Y2H) assay

The *BrRGF6* coding sequence with the termination codon removed was amplified (using the primers shown in Table S1) and cloned into the *Ned*I and *Bam*HI sites of vector pGBKT7 to obtain the bait vector pGBKT7-BrRGF6. pGBKT7-BrRGF6 or pGBKT7-T (negative control) were transformed into the yeast Y2H strain and spread on SD-Trp (SDO) for the toxicity assay. pGBKT7-BrRGF6 + pGADT7-T were co-transfected into the yeast Y2H strain, and then spread on SD/−Trp/−Leu/X-a-gal/AbA (DDO/X/A) for self-activation detection (with pGBKT7–53 + pGADT7-T as the positive control).

Using Y2H, proteins that interact with BrRGF6 were screened from the cDNA library of Chinese cabbage roots infected with *Plasmodiophora brassicae* [21] by the mating method following the instructions for construction of Mate Plate Yeast Two-hybrid Library (Clontech, Mountain View, CA, USA). The hybrid solution was spread on 50 SD/−Trp/−Leu/−his (TDO) plates to observe their growth. Well-growing colonies were re-plated on the new TDO medium to observe their growth. The positive clones from secondary screening were sequenced (Preintel bio, Shanxi, China), and their function was analyzed by NCBI BLAST (Basic Local Alignment Search Tool (nih.gov)). The full-length cDNA sequence encoding the candidate interaction protein (BrNF-YC), which was selected from the Y2H library, was amplified using the primers shown in Table S1 and cloned into the *Eco*RI and *Bam*HI sites of vector pGADT7-T as the prey vector and used to verify its interaction with pGBKT7-BrRGF6 by conjugative transfer (Co-transfer). 5 μL of the co-transfer strains at different dilution concentrations (× 10^0^, × 10^−1^, × 10^−2^, × 10^−3^) were dropped on the SD/−Trp/−Leu/-His and photographed after incubation at 30°C for 4 days. At the same time, pGADT7-T + pGBKT7–53 and pGADT7-T + pGBKT7-Lam were used as positive and negative controls, respectively.

### Verification of the binding between BrNF-YC and ProBrRGF6 by yeast one-hybrid (Y1H) analysis

The upstream promoter of the *BrRGF6* gene (*PBrRGF6*) was amplified using leaf DNA of “SN742” (using the primers shown in Table S1), and the promoter elements were analyzed using Plantcare (http://bioinformatics.psb.ugent.be/webtools/plantcare/html/). *PBrRGF6* was amplified (using the primers shown in Table S1) and cloned into the *Hind*III and *Xho*I sites of vector pAbAi to obtain recombinant vector p*P_BrRGF6_-*AbAi. Next, p*P_BrRGF6_-*AbAi was linearized using *Bst*bI (NEB, Beijing, China) and transferred into the Y1H strain. The positive yeast Y1H-p*P_BrRGF6_-*AbAi was transferred to SD/-Ura/AbA (50–500 ng/mL) and screened for the minimum inhibitory concentration of AbA (AbA^r^) in relation to yeast growth for a Y1H assay.

The recombinant vector pGADT7-BrNF-YC was transferred to yeast strain Y1H-p*P_BrRGF6_-*AbAi. 5 μL of the strain at different dilution concentrations (× 10^0^, × 10^−1^, × 10^−2^, × 10^−3^) was dropped on SD/−Leu and SD/−Leu/AbA^r^ media to verify the binding between BrNF-YC and *PBrRGF6*. At the same time, pGADT7-T was used as a negative control..

### GUS activity analysis

For GUS activity analysis, recombinant vectors pBI101-*PBrRGF6* (reporter vector) and pRI101-BrNF-YC (effector vector) were constructed (using the primers shown in Table S1), and pRI101vector (effector vector) was used as the control. These vectors were transferred into *A. tumefaciens* strain GV3101, respectively, and cultured to OD_600_ = 1.0 at 28°C. The cells were harvested and resuspended in medium (1 M MgCl_2_, 100 mM acetosyringone, and 1 M MES, pH = 5.6). The reporter and effector constructs were co-infiltrated into tobacco leaves, and the infiltrated tobacco plants were kept in the dark at room temperature for 3 d. GUS activity was determined by referring to the methods of Li et al [22].

Specific primers of *BrNF-YC* for qRT-PCR were designed (Table S1), and the expression pattern of *BrNF-YC* in Chinese cabbage roots was further analyzed.

### Gene function verification by virus induced gene silencing (VIGS)

To further study the function of *BrRGF6* and *BrNF-YC* in response to *Plasmodiophora brassicae* infection, a 300 bp *BrRGF6*-specific fragment and a 300 bp *BrNF-YC*-specific fragment were cloned into virus vector pTRV2, separately, according to a previously described method [22]. pTRV1, pTRV2, pTRV2-*BrRGF6* and pTRV2-*BrNF-YC* were transferred into *A. tumefaciens* strain GV3101 separately using the freeze–thaw method [23] and cultured to OD_600_ = 1.0 at 28°C. The cells were collected and resuspended in infection solution (10 mM MgCl_2_, 10 mM MES and 150 mM Acetosyringone, PH = 5.7). After incubation at room temperature for 3–5 hours in the dark, the above infection solution containing vectors pTRV2, pTRV2-*BrRGF6*, or pTRV2-*BrNF-YC* were mixed respectively with the infection solution containing pTRV1 vector (1:1, vol/vol). Germinated “ECD05” seeds were infected with above mixed infection solution using the vacuum method [24]. Plants carrying pTRV1 + pTRV2 (TRV::00) was used as controls, plants carrying pTRV1 + pTRV2-*BrRGF6* (TRV::*BrRGF6*) or pTRV1 + pTRV2-*BrNF-YC* (TRV::*BrNF-YC*) were set as the experimental groups, and 60 seeds were infected in each group (the experiment was repeated three times). The seeds treated with the infection solution containing GV3101 were seeded in sterilized soil containing 50% matrix, and grown in a 25°C incubator with a 16 h light/8 h dark cycle. Roots samples were taken at 14^th^, 21^st^, and 28^th^ d after infection to detect the silencing efficiency. RNA from roots samples were extracted and gene expression was analyzed using qRT-PCR. When the gene was confirmed as silenced, the roots were inoculated with *Plasmodiophora brassicae*. In detail, the *Plasmodiophora brassicae* suspension was adjusted to OD_600_ = 1.0, and 1 mL was injected into the soil around the root of each plant (30 plants in each experimental group were inoculated). The disease index was investigated at 25–35 days after inoculation with *Plasmodiophora brassicae*. The disease indexes of the silenced lines were graded, then the incidence rate and disease index were calculated following the methods of LV et al [15]. qRT-PCR was used to analyze the gene expression in the roots of the silenced plants not infected with *Plasmodiophora brassicae* and the phenotypes of all experimental plants were observed. Five representative plants of each experimental group were selected for root measurement and statistics. Duncan’s test was used to identify significant difference of root length between different groups.

### Gene function verification by *agrobacterium* mediated transient overexpression transformation

The coding sequence with termination codon removed of *BrRGF6* or *BrNF-YC* was respectively constructed into pSuper1300-GFP (pSuper::GFP) vectors (using the primers shown in Table S1) to construct pSuper::*BrRGF6*-GFP and pSuper::*BrNF-YC*-GFP recombinant vectors, and *Agrobacterium* mediated transient overexpression transformation was carried out on Chinese cabbage in the same way as VIGS. At the same time, pSuper::GFP was used as the negative control. Due to different vectors, samples were taken on the 7^th^, 14^th^ and 21^st^ days after treatment respectively to accurately detect the best efficiency of transient overexpression, and *Plasmodiophora brassicae*s inoculation was carried out when the gene expression increased significantly. At the same time, the positive overexpressed plants were selected by observing the GFP signal in the transient overexpression plant leaves using Tanon-5200 chemiluminescence imaging system (Tanon, Shanghai, China), the plant leaves that were not transiently transformed were used as the control. Observe the growth of the plant after inoculation, and identify the disease index when the plant has obvious root swelling symptom.

## Acknowledgments

We would like to thank the English scientists of Elixigen Company (Huntington Beach, California) for English language editing. This work was supported by grants from the National Natural Science Foundation of China [grant number 31972412; 32272717].

## Author contributions

Conceptualization, WG, JZ and RJ; Data curation, WG and JZ; Formal analysis, WG, ZJ and RJ; Funding acquisition, RJ; Investigation, JZ, YW, and WG; Methodology, JZ and WG; Supervision, RJ and HF; Writing-original draft, WG and ZJ; Writing-review & editing, WG and RJ. All authors have read and agreed to the published version of the manuscript.

## Data availability statement

All data supporting the findings of this study are available within the paper and within its supplementary data.

## Conflicts of interest

The authors declare that they have no conflict of interest.

## Supplementary data

Supplementary data is available at *Horticulture Research Journal* online.

## Supplementary Material

Web_Material_uhac292
