# Supplementary material for: The root meristem growth factor BrRGF6 positively regulates Chinese cabbage to infection of clubroot disease caused by Plasmodiophora Brassicae
Source: Hortic Res. 2022 Dec 30;10(3):uhac292. doi: 10.1093/hr/uhac292 (PMC10018783; doi:10.1093/hr/uhac292)
Supplement: Web_Material_uhac292 [file web_material_uhac292.zip › supplement file.docx]

**Table S1. Primers used in this study**

| **Name** | **Sequence (5’-3’)** | | **Purpose** |
| --- | --- | --- | --- |
| ***BrRGF6*** | Forward | TATCATATGTATTGCCCTGTGAAGCG | cDNA sequence amplification |
|  | Reverse | TTAGGATCCAGCCTTCTCGTTGTGGATGG |  |
| ***BrRGF6-*sub** | Forward | CGCCACTAGTCATATGATGGTGTTTTGTTTCATTCTTCTGC | Subcellular localization |
|  | Reverse | GAGCGGTACCGGATCCAGCCTTCTCGTTGTGGATGG |  |
| ***BrRGF6*-qRT** | Forward | CGTTGGTTGTGCCACTGCT | qRT-PCR |
|  | Reverse | CATCTCCCCCCGTCTCCTCT |  |
| ***BrActin*** | Forward | ATCTACGAGGGTTATGCT | qRT-PCR |
|  | Reverse | CCACTGAGGACGATGTTT |  |
| ***BrRGF6*-*sit****u* | Forward | ACAAGCTTGCATGCAAGCTTATGTATTGCCCTGTGAAGCG | *in situ* RNA hybridization |
|  | Reverse | CGGAATTCGAGCTGGATCCAGCCTTCTCGTTGTGGATGG |  |
| ***rgf6*** | LP | ACCATTCATTGTTCCTTGCAG | identification of *Arabidopsis* mutants |
|  | RP | ATGATAATGAAGGACCCCTCC |  |
|  | LB | ATTTTGCCGATTTCGGAAC |  |
| **18S** | Forward | CGGCTACCACATCCAAGGAA | qRT-PCR |
|  | Reverse | GCTGGAATTACCGCGGCT |  |
| ***AtRGF6*- qPCR** | Forward | GCTCTAGTCTCATCTCCCTGTA | qRT-PCR |
|  | Reverse | CACTCCTCAAAGAGCAAGACA |  |
| **AtNF-YC-qPCR** | Forward | GCGAGCGGTGTTCCTTATTA | qRT-PCR |
|  | Reverse | AGAAGGAGGCTGAGCATAAAC |  |
| **BrRGF6-BD** | Forward | AGGAGGACCTGCATATGGTGTTTTGTTTCATTCTTCTGC | Yeast two-hybrid analysis |
|  | Reverse | GCAGGTCGACGGATCCAGCCTTCTCGTTGTGGATGGG |  |
| ***PBrRGF6*** | Forward | TCCCTAGATCTCTCTCGCTGGATAT | promoter region amplification |
|  | Reverse | CATCGAAGAGAGCCCACGC |  |
| **P-*P_BrRGF_*_6_-AbAi** | Forward | ATGAATTGAAAAGCTTTCCCTAGATCTCTCTCGCTGGATAT | Yeast one-hybrid analysis |
|  | Reverse | AGAGCACATGCCTCGAGCATCGAAGAGAGCCCACGC |  |
| **BrNF-YC-AD** | Forward | GGAGGCCAGTGAATTCATGGACAACAGCAACCAGCAATCA | Yeast one-hybrid analysis |
|  | Reverse | CGAGCTCGATGGATCCACACTTGCCCGTCAAGATTCCCT |  |
| **pBI-*PBrRGF6*** | Forward | TGATTACGCCAAGCTTTCCCTAGATCTCTCTCGCTGGATAT | GUS activity analysis |
|  | Reverse | GACCACCCGGGGATCCCATCGAAGAGAGCCCACGC |  |
| **BrNF-YC-PRI** | Forward | CACTGTTGATACATATGGACAACAGCAACCAGCAATCA | GUS activity analysis |
|  | Reverse | TGTTGATTCAGAATTCACACTTGCCCGTCAAGATTCCCT |  |
| **BrRGF6-pSuper** | Forward | CCAAATCGACTCTAGATGTATTGCCCTGTGAAGCGTGG | Transient transformation |
|  | Reverse | CCCTTGCTCACCATGAGCCTTCTCGTTGTGGATGGGAGGC |  |
| **BrNF-YC- pSuper** | Forward | CCAAATCGACTCTAGATGGACAACAGCAACCAGCAATCAT | Transient transformation |
|  | Reverse | CCTTGCTCACCATGCACTTGCCCGTCAAGATTCCCT |  |

**Table S2. Disease incidence and index of *BrRGF6* and *BrNF-YC* silenced Chinese cabbage infected by *P. brassicae***

Note: DI, Disease index. Data shown represent mean ± SD (n = 3). p < 0.05, according to Duncan’s multiple range test.

| **Group** | **Incidence (%)** | **DI** |
| --- | --- | --- |
| TRV::00 | 70.00 ± 3.33^a^ | 43.89 ± 1.73^a^ |
| TRV::BrRGF6 | 36.67 ± 3.33^b^ | 17.78 ± 0.96^b^ |
| TRV::BrNF-YC | 36.11 ± 5.36^b^ | 19.99 ± 3.00^b^ |

**Table S3. Investigation of *BrRGF6* and *BrNF-YC* silenced Chinese cabbage root length (cm)**

Note: Data shown represent mean ± SD (n = 5). p < 0.05, according to Duncan’s multiple range test.

| **Group** | **Root length (cm)** |
| --- | --- |
| TRV::00 | 2.673 ± 0.021^b^ |
| TRV::BrRGF6 | 4.237 ± 0.029^a^ |
| TRV::BrNF-YC | 4.240 ± 0.037^a^ |

**Table S4. Disease incidence and index of *BrRGF6* and *BrNF-YC* overexpression Chinese cabbage infected by *P. brassicae***

Note: DI, Disease index. Data shown represent mean ± SD (n = 3). p < 0.05, according to Duncan’s multiple range test.

| **Group** | **Incidence (%)** | **DI** |
| --- | --- | --- |
| pSuper::GFP | 64.00 ± 1.91^b^ | 24.85 ± 1.65^b^ |
| pSuper::*BrRGF6*-GFP | 71.91 ± 1.98^a^ | 42.59 ± 9.37^a^ |
| pSuper::*BrNF-YC-*GFP | 70.00 ± 1.98^a^ | 37.77 ± 2.41^a^ |

**Table S5. Investigation of *BrRGF6* and *BrNF-YC* overexpression Chinese cabbage root length (cm)**

Note: Data shown represent mean ± SD (n = 5). p < 0.05, according to Duncan’s multiple range test.

| **Group** | **Root length (cm)** |
| --- | --- |
| TRV::00 | 3.847 ± 0.338^a^ |
| TRV::BrRGF6 | 2.487 ± 0.246^b^ |
| TRV::BrNF-YC | 3.117 ± 0.767^ab^ |


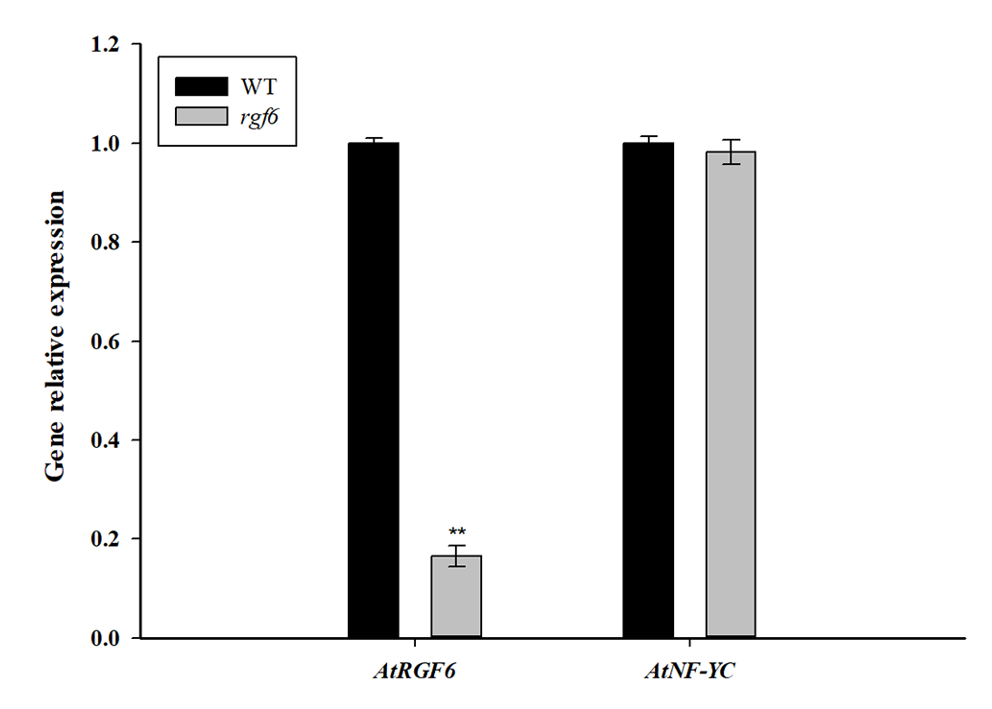


**Fig. S1 Analysis of gene expression pattern in *Arabidopsis rgf6* mutant.** Data shown represent mean ± SD (n = 3). p < 0.05, according to a t‑test.


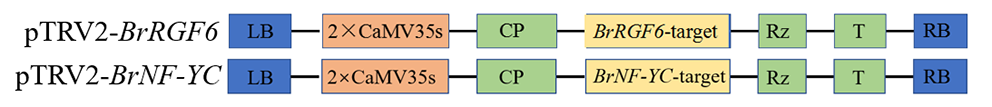


**Fig. S2 Structure diagram of pTRV2-*BrRGF6* and pTRV2-*BrNF-YC* in VIGS.**
